# The Change in Fatty Acids and Sugars Reveals the Association between Trifoliate Orange and Endophytic Fungi

**DOI:** 10.3390/jof7090716

**Published:** 2021-08-31

**Authors:** Lu-Lu Meng, Rui-Cheng Liu, Liu Yang, Ying-Ning Zou, Anoop Kumar Srivastava, Kamil Kuča, Abeer Hashem, Elsayed Fathi Abd_Allah, Bhoopander Giri, Qiang-Sheng Wu

**Affiliations:** 1 College of Horticulture and Gardening, Yangtze University, Jingzhou 434025, China; lulu_meng1997@163.com (L.-L.M.); 202072809@yangtzeu.edu.cn (R.-C.L.); 2536405387@qq.com (L.Y.); 500406@yangtzeu.edu.cn (Y.-N.Z.); 2 ICAR-Central Citrus Research Institute, Nagpur 440033, Maharashtra, India; aksrivas2007@gmail.com; 3 Department of Chemistry, Faculty of Science, University of Hradec Králové, 50003 Hradec Králové, Czech Republic; kamil.kuca@uhk.cz; 4 Botany and Microbiology Department, College of Science, King Saud University, P.O. Box 2460, Riyadh 11451, Saudi Arabia; habeer@ksu.edu.sa; 5 Plant Production Department, College of Food and Agricultural Sciences, King Saud University, P.O. Box 2460, Riyadh 11451, Saudi Arabia; eabdallah@ksu.edu.sa; 6 Department of Botany, Swami Shraddhanand College, University of Delhi, Delhi 110036, India; bhoopg@yahoo.com

**Keywords:** carbohydrate, citrus, endophytes, fatty acid, symbiosis

## Abstract

Endophytes have the ability to improve plant nutrition alongside their agronomic performance, among which arbuscular mycorrhizal fungi provide the most benefits to their host. Previously, we reported for the first time that an arbuscular mycorrhizal-like fungus *Piriformospora indica* had the ability to colonize roots of trifoliate orange (*Poncirus trifoliata*) and conferred positive effects on nutrient acquisition. Present study showed the changes in fatty acids and sugars to unravel the physiological and symbiotic association of trifoliate orange with *P*. *indica* and an arbuscular mycorrhizal fungus, *Funneliformis mosseae* singly or in combination. All the endophytic fungi collectively increased fructose, glucose, and sucrose content in leaves and roots, along with a relatively higher increase with *P*. *indica* inoculation than with *F*. *mosseae* alone or dual inoculation. Treatment with *P*. *indica* increased the concentration of part unsaturated fatty acids such as C18:3N6, C20:2, C20:3N6, C20:4N6, C20:3N3, C20:5N3, C22:1N9, and C24:1. Additionally, *P*. *indica* induced the increase in the concentration of part saturated fatty acids such as C6:0, C8:0, C13:0, C14:0, and C24:0. *F*. *mosseae* hardly changed the content of fatty acids, except for increase in C14:0 and C20:5N3. Double inoculation only reduced the C21:0, C10:0, C12:0, C18:3N3, and C18:1 content and increased the C20:5N3 content. These endophytic fungi up-regulated the root *PtFAD2, PtFAD6, PtΔ9*, and *PtΔ15* gene expression level, coupled with a higher expression of *PtFAD2* and *PtΔ9* by *P*. *indica* than by *F*. *mosseae*. It was concluded that *P*. *indica* exhibited a stronger response, for sugars and fatty acids, than *F*. *mosseae* on trifoliate orange. Such results also reveal the *Pi* (an *in vitro* culturable fungus) as a bio-stimulator applying to citriculture.

## 1. Introduction

Terrestrial plants are reported to form mutual symbioses with endophytic fungi in roots, without causing any damage to host plants [1,2]. Amongst endophytic fungi, arbuscular mycorrhizal (AM) fungi predominantly occurring in the soil have the ability to colonize roots of more than 72% of terrestrial plants and, thus, form arbuscular mycorrhizas in the roots [3]. In such a mutualistic relationship, host plants provide the required substances to AM fungi for their growth and, in reciprocation, AM fungi facilitate water and nutrient acquisition of the host [4,5]. As a result, host plants offer mycorrhizal-C pool in the roots, which inevitably affects the extent of carbohydrate accumulation in the plant roots [6]. However, plant roots also utilize a substantial amount of carbohydrates for their growth and functions [7,8]. Within endophytes, the AM-like fungus, *Piriformospora indica* (*Pi*) of the Sebacinales, Basidiomycota, is extensively reported to colonize many plants for their growth promotion and imparting tolerance against varied biotic and abiotic stress [9,10,11,12].

The role of fatty acids (FAs) in response to mycorrhization for imparting drought tolerance to host plants is well documented [5]. FAs, as a main component of cell membrane, are divided into saturated fatty acids (SFAs) and unsaturated fatty acids (UFAs). Recent studies showed that AM fungi obtained FAs from host plants [4,13], further supporting the operation of the AM-regulated lipid pathway into the arbuscule-containing cells of host plants [13]. In *Rhizophagus irregularis*-mediated responses, myristic acid (C14:0) was used as a source of FAs for the hyphal growth of germinated spores, and C14:0 also induced the formation of secondary spores with features of a branched absorbing structure and dense coils [14]. On the other hand, (*S*)-12-methyltetradecanoic acid (a methyl-branched chain FA) released from bacterial cultures induced the branching of hyphae originated from mother spores leading to the formation of secondary spores, and palmitoleic acid (a SFA type) aided in the production of a higher number of secondary spores than the bacterial (*S*)-12-methyltetradecanoic acid [3]. One of our earlier studies showed that AM fungal colonization distinctly increased contents of UFAs in trifoliate orange by up-regulating the root *FA desaturase* (*FAD*) gene (e.g., *FAD2* and *FAD6*) under either optimum water or soil water deficit conditions [5], suggesting further that the composition and saturation of FAs are governed by AM fungi under soil drought conditions. Hua et al. [15] demonstrated that the host metabolic compounds and metabolite pathways were reprogrammed following symbiosis upon inoculation with endophytic fungi. However, little is known about the effects of endophytic fungi on the composition and saturation of FAs synthesized by host plants. 

Citrus is globally one of the most important fruit crops and displays lesser root hair growth in the field, but is considered highly dependent on AM fungi [16]. In vitro propagation of AM fungi is the major drawback, while *Pi* isolated in 1996 displayed an *in vitro* propagation ability without roots being able to colonize citrus [10,17]. 

The present study aims to study the response of trifoliate orange (*Poncirus trifoliata* L. Raf., a widely used citrus rootstock) to being colonized by an AM fungus (*Funneliformospora mosseae*, *Fm*) versus an AM-like fungus *Pi*: (i) changes in composition and saturation levels of FAs; (ii) changes in root sugar; (iii) expression levels of FA desaturase genes. Such work would reveal the symbiotic association between trifoliate orange and endophytic fungi.

## 2. Materials and Methods

### 2.1. Endophytic Fungi

*Fm* was provided by the Bank of Glomales in China. The AM fungus strain was propagated using *Trifolium repens* L. as a host plant for 16 weeks under potted conditions. The 80 g inoculum of *Fm* containing spores (20 spores/g), mycelia, and AM fungi-colonized root fragments was added into the pot as AM fungal treatment at the time of transplanting of trifoliate orange. 

*Pi* was propagated following the protocol of Yang et al. [17]. A 5 × 5 mm fungal mass was activated twice at 30 °C for 7 days on solid culture medium of potato dextrose agar. The activated mycelium was proliferated on the liquid culture medium of potato dextrose broth under dark for 7 days. Spore suspensions were collected, washed with 0.05% Tween 20 in ddH_2_O, and centrifuged at 4000× *g*/min for 7 min. The collected spores were mixed with distilled water at a ratio of 1:20, and the number of spores was determined colorimetrically at 600 nm, reaching the concentration of 3.27 × 10^8^ CFU/mL. 

### 2.2. Experimental Setup

The three 4-leaf-old trifoliate orange seedlings grown in autoclaved sands were transplanted into a 1.4 L pot, in which autoclaved (0.11 MPa, 2 h) growth substrates (1.5 kg) consisting of coarse-textured loam soil and sand (5:3, *v*/*v*) were supplied. A single *Fm* inoculum of 80 g per pot was applied at transplanting, and a single inoculation of *Pi* with 20 mL spore suspension was performed. The dual inoculation of *Fm* and *Pi* consisted of an 80 g inoculum of *Fm* and 20 mL spore suspension of *Pi* together during plant transplanting. Non-fungi-treated plants (control) were inoculated without any of the two fungi materials as the control. These treated plants were grown for 140 days under controlled environmental conditions, as previously described by Yang et al. [17].

The experiment was, therefore, arranged with four inoculations with single *Fm*, single *Pi*, dual *Fm* + *Pi*, and non-fungi control. Each treatment had ten replicates in a completely randomized design.

### 2.3. Measurement of Root Fungal Colonization and Plant Biomass

The roots were removed from the growing medium. A segment taken from the middle part of the root was carefully washed, cut into 1 cm long root segments, cleared with 10% KOH solutions at 95 °C for 1.5 h, followed by bleaching with 10% H_2_O_2_ solution for 15 min, acidified with 0.2 mol/L HCl for 1 h, and, finally, stained with 0.05% (*w*/*v*) trypan blue in lactophenol for 3 min [18]. The root fungal colonization degree was computed as the percentage of fungal colonized root number versus total observed root number as suggested by Yang et al. [17]. The shoots and roots were dried in oven (75 °C, 48 h) and weighed to record their biomass.

### 2.4. Measurement of Sugars and FA Contents

The fructose, glucose, and sucrose concentrations of leaves and roots were determined using the colorimetric method suggested by Wu et al. [6]. Root FAs were extracted and quantified according to the protocol outlined by Wu et al. [5], with an external standard with the NU-CHEK-PREP 37 fatty acid methyl ester-mixed solution (1000 μg/mL). 

### 2.5. Relative Expression of Root FA Desaturase Genes

Root samples were ground with liquid nitrogen, and total RNA was extracted with the help of Plant RNA Extraction Kit (Takara Bio. Inc.). The RNA was reverse-transcribed into cDNA using a PrimeScript^TM^ RT reagent kit with cDNA eraser. The sequences of four FA desaturase genes, including *FA desaturase 2* (*PtFAD2*), *FA desaturase 6* (*PtFAD6*), *Δ9 FA desaturase* (*PtΔ9*), and *Δ15 FA desaturase* (*PtΔ15*) were obtained based on the sweet orange database (http://citrus.hzau.edu.cn/, 10 October 2020), and specific primer sequences (Table 1) were designed using the Primer Premier 5.0. The reaction system and condition of qRT-PCR were carried out according to the method described by Wu et al. [5]. Relative gene expression was worked out according to the method suggested by Livak and Schmittgen [19], based on *β-actin* as a house-keeping gene [20]. The measured transcripts were normalized to the relative expression value in non-fungi-inoculated plants.

### 2.6. Data Analysis

The data generated by the experiment were analyzed with one-factor analysis of variance through the SAS software. Duncan’s multiple range tests were used at the 0.05% level to compare the significant difference amongst the treatments.

## 3. Results

### 3.1. Root Colonization after Inoculation of Endophytic Fungi

**Figure 1:**
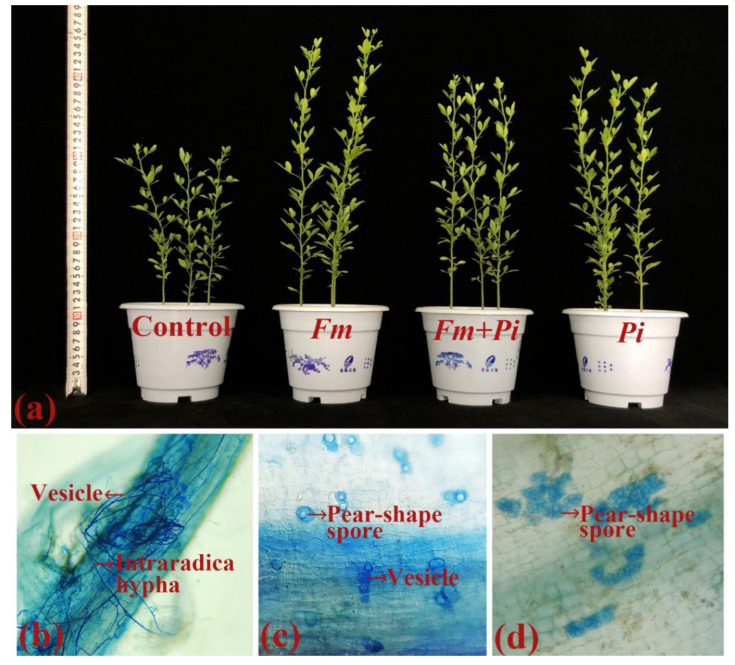
Plant growth response (**a**) and root fungal colonization (**b**–**d**) of trifoliate orange seedlings by *Funneliformis mosseae* (*Fm*) and *Piriformospora indica* (*Pi*) singly and in combination. (**a**) Plant growth change after inoculation; (**b**) intraradical hyphae, vesicles, and arbuscules in *Fm*-inoculated roots; (**c**) pear-shaped spores and vesicles in dual inoculated roots by *Fm* + *Pi*; (**d**) groups of pear-shaped spores in *Pi*-inoculated roots.

**Figure 2:**
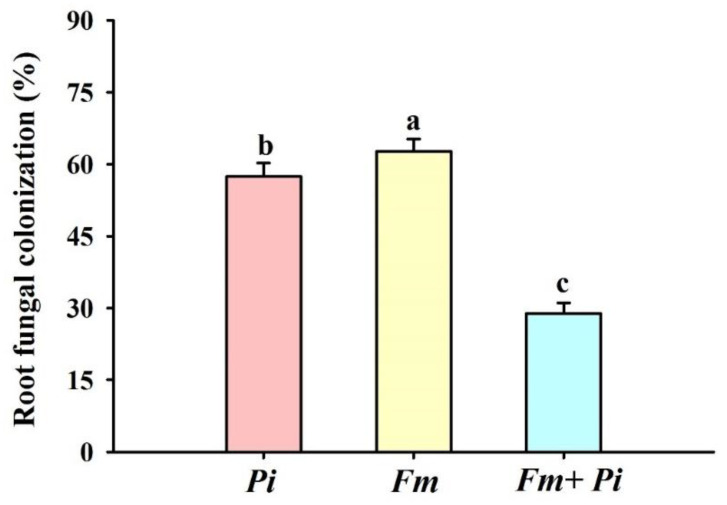
Effects of *Piriformospora indica* (*Pi*) and *Funneliformis mosseae* (*Fm*) singly and in combination on the root fungal colonization in trifoliate orange seedlings. Data (means ± SD, *n* = 10) followed by different letters above the bars indicate significant differences (*p* < 0.05) between treatments.

No fungal colonization was found in the non-inoculated trifoliate orange seedlings, while fungal colonization (28.82–62.68%) was present in the roots of *Fi*, *Pi*, and *Fi* + *Pi*-inoculated seedlings, respectively (Figure 1b–d and Figure 2). The root fungal colonization was observed in a decreasing order of *Fm* > *Pi* > *Fm* + *Pi* (Figure 2).

### 3.2. Effects of Endophytic Fungi on Biomass Production

**Figure 3:**
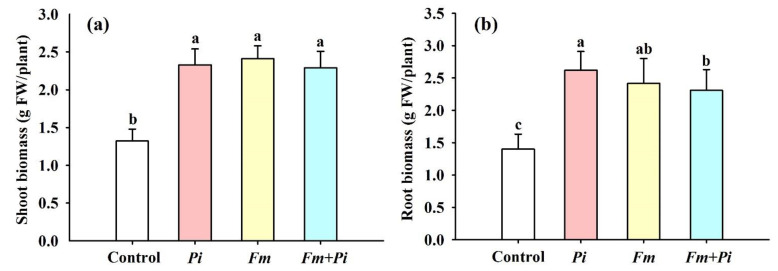
Effects of *Piriformospora indica* (*Pi*) and *Funneliformis mosseae* (*Fm*) singly and in combination on shoot (**a**) and root (**b**) biomass of trifoliate orange seedlings. Data (means ± SD, *n* = 10) followed by different letters above the bars indicate significant differences (*p* < 0.05) between treatments.

Single as well dual fungal inoculation collectively improved shoot and root biomass (Figure 1a and Figure 3a,b). Compared with the control (non-fungi-inoculated treatment), endophytic fungi inoculation significantly improved the shoot biomass by 77%, 83%, and 74%, respectively, with *Pi*, *Fm*, and the combination of *Fm* + *Pi* (Figure 3a). The root biomass was increased by 87%, 73%, and 65% with *Pi*, *Fm*, and *Fm* + *Pi* (Figure 3b), respectively, compared with the control. In brief, the improvement of shoot biomass did not show significant differences between endophytic fungal treatments, but the differences of the root biomass improvement were significant, showing a decreasing trend with *Pi > Fm > Fm + Pi*.

### 3.3. Effects of Endophytic Fungi on Sugar Concentrations of Leaves and Roots

*Pi* increased the concentration of sucrose, glucose, and fructose by 41%, 159%, and 16%, respectively, in leaves with the corresponding increase of 124%, 65%, and 23% in roots over the control (Table 2), while *Fm* increased sucrose, glucose, and fructose concentrations by 25%, 135%, and 11%, respectively, in leaves with the corresponding increase of 37%, 46%, and 21% in roots, compared with the control. The dual inoculation increased sucrose, glucose, and fructose concentrations in leaves by 13%, 29%, and 10%, respectively, with the corresponding increase of 104%, 50%, and 15% in roots, compared with the control. In addition, the increase in sugar was distinctly higher with *Pi* than with *Fm* and *Fm* + *Pi*, independent of leaves and roots. 

### 3.4. Effects of Endophytic Fungi on FAs Contents in Roots

Fifteen SFAs and fourteen UFAs were identified in roots of trifoliate orange, and C11:0, C14:1, C15:1, C22:2, and C22:6N3 were not identified (Table 3). The major SFAs in trifoliate orange comprised of C16:0 and C18:0, accounting for 57–58% and 36–37%, respectively, out of total SFAs, while the important UFAs in roots were C18:1, C18:2, and C18:3N3, amounting to 8–18%, 55–56%, and 15%, respectively, out of total UFAs in roots.

Single inoculation with *Pi* significantly increased part UFAs concentrations such as C18:3N6, C20:2, C20:3N6, C20:4N6, C20:3N3, C20:5N3, C22:1N9, and C24:1 by 64%, 51%, 103%, 35%, 27%, 190%, 41%, and 69%, while it did not affect C16:1, C17:1, C18:1, C18:2, C18:3N3, and C20:1 concentrations. Single inoculation with *Fm* had no significant effect on the concentration of UFAs except that the C20:5N3 of UFAs, which was significantly increased by 158%. In addition, the double inoculation of *Fm* and *Pi* triggered the increase in C20:5N3 by 331% and the decrease in C18:1 by 28% and C18:3N3 by 22%, coupled with no change in other UFAs. 

As for SFAs, single inoculation with *Pi* distinctly increased the C6:0, C8:0, C13:0, C14:0, and C24:0 concentration by 45%, 39%, 12%, 31%, and 36%, respectively, whereas it had no significant effect on other SFAs. *Fm* inoculation did not have any effects on all SFA concentrations, except the significant increase in C14:0 by 17%. Dual inoculation of *Fm* and *Pi* only reduced the C10:0, C12:0, and C21:0 concentration by 25%, 17%, and 18%, respectively. The content of all detected SFAs (except C17:0) almost showed a decreasing trend of *Pi* > *Fm* > *Fm* + *Pi* among endophytic fungal inoculation treatments.

### 3.5. Effects of Endophytic Fungi on Relative Expression of FA Desaturase Genes

**Figure 4:**
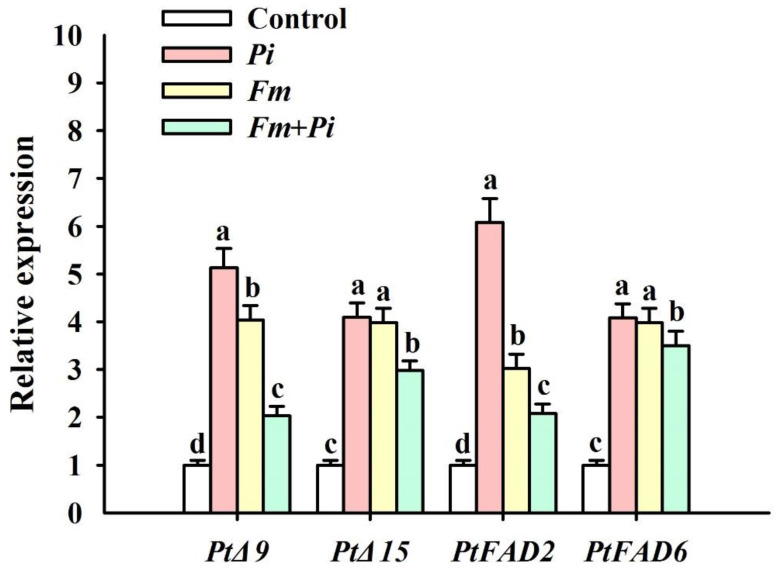
Effects of *Piriformospora indica* (*Pi*) and *Funneliformis mosseae* (*Fm*) singly and in combination on the relative expression levels of four FA desaturase genes in roots of trifoliate orange seedlings. Data (means ± SD, *n* = 3) followed by different letters above the bars indicate significant differences (*p* < 0.05) between treatments.

The expression level of *PtΔ9*, *PtFAD2*, *PtFAD6*, and *PtΔ15* was increased by 413%, 298%, 508%, and 298%, respectively, with *Pi* inoculation compared with the control (Figure 4). *Fm* inoculation significantly induced the expression of *PtΔ9*, *PtFAD2*, *PtFAD6*, and *PtΔ15* by 304%, 309%, 202%, and 308%, respectively, and dual inoculation of *Fm* + *Pi* registered a 103%, 198%, 108%, and 250% higher expression level of *PtΔ9*, *PtFAD2*, *PtFAD6*, and *PtΔ15*, compared with the control. In brief, the relative expression levels of *PtΔ9*, *PtΔ15*, *PtFAD2*, and *PtFAD6* genes showed a decreasing trend of *Pi* > *Fm* > *Fm* + *Pi* > control among the four treatments.

## 4. Discussion

In this study, we observed that three endophytic fungi inoculations displayed a differential magnitude of root fungal colonization in the decreasing order of *Fm* > *Pi* > *Fm* + *Pi*, indicating that trifoliate orange is preferentially colonized by *Fm*. Two endophytic fungi (*Fm* and *Pi*) colonized the roots of trifoliate orange when inoculated separately, but their dual inoculation of *Fm* + *Pi* failed to register the same magnitude of root fungal colonization. It has been documented that the proliferation of *Pi* in roots needs dead cells of host plants [21]. Thus, when *Fm* and *Pi* were co-present in the roots of trifoliate orange, the cell death induced by *Pi* may have reduced the colonization of living cells by *Fm*. More work needs to be started around the change of cells colonized by both AM fungi and *Pi*. 

AM fungi are reported to acquire most of the carbohydrates, especially hexose, from the host root, transforming them into typical fungal carbohydrates, since AM fungi operate through a typical process of mutualistic symbiosis, depending upon the host plant to be able to transport the photosynthetic products for their multiplication [22]. In this process, AM fungi only absorb and utilize small molecules as glucose, while sucrose and other macromolecular substances need to be cleaved into glucose and fructose before being absorbed and utilized by mycorrhizal plants [23]. Our study showed that a higher sucrose content in the leaves of mycorrhizal plants was beneficial for the downward movement of sucrose in phloem tissue. Thus, sucrose in roots of mycorrhizal plants is accountable to the cleavage through catabolic enzymes into hexose for onward mycorrhizal development, or the development of a more effective root morphology of mycorrhizal plants, thereby, consuming more sucrose for respiration as a pre-requisite of AM fungi to be more efficacious [24]. *Fm* inoculation increased the glucose content, due to the existence of a better carbon pool in mycorrhizal roots requiring a comparatively higher amount of sucrose to move from leaves to roots; thus, reducing the sucrose cleavage in leaves and increasing the glucose content as a cause-and-effect relationship in the process of AM symbiosis. Amongst different carbohydrates, sucrose has the ability to stimulate a lateral root formation [25]. Glucose, as an important regulator of plant growth and development, participates in the gibberellin and cytokinin signaling pathway [26]. The higher the carbohydrate content in the roots of endophytic fungi-inoculated trifoliate orange, the more beneficial it is to the better growth of roots. Among *Fm* and *Pi*, we found a higher increase in fructose, glucose, and sucrose in leaves and roots by *Pi* inoculation than by *Fm* inoculation or dual inoculation, indicating that *Pi* triggered a stronger demand for C and C pools in roots than the two fungi inoculations. In order to understand the relationship between endophytic fungi and carbohydrate metabolism, we need to further bring the role of endogenous hormones, especially abscisic acid, gibberellins, and cytokinin, to unravel the underlying mechanisms operating in the host-endophytic fungi interaction. 

Our results showed an increase in many UFA concentrations such as C18:3N6, C20:2, C20:3N6, C20:4N6, C20:3N3, C20:5N3, C22:1N9, and C24:1 after inoculation with single *Pi*, thereby leading to a higher degree of unsaturation in the *Pi*-inoculated plants, which would maintain a higher cell membrane fluidity and lower innate immunity against exogenous fungal colonization [27]. In contrast, *Fm* hardly changed the content of any UFAs (except the increase in C20:5N3). Thus, this appears to imply that roots of trifoliate orange were more susceptible to *Pi* colonization than *Fm* colonization. AM fungi (*Fm*) did not change any SFA contents (except the increase in C14:0), and *Pi* inoculation also increased five SFA (including C14:0) contents. A common feature of both fungi was the increased content of the SFA C14:0 as well as the UFA C20:5N3. Sugiura et al. [14] also reported that C14:0 could favor the mycelial growth of budding mycorrhizal fungal spores as well as the formation of secondary spores. Therefore, in FAs, C14:0 could be used as a characterizer to evaluate the developmental status of endophytic fungi. However, the double inoculation of *Fm* and *Pi* did not induce any significant change in the C14:0 concentration, indicating that the growth of both fungi consumed excess C14:0 and, therefore, triggered no change in C14:0 in roots of the host. An increase in C20:5N3 under fungal colonization conditions was found in *Medicago truncatula* plants inoculated with *Glomus intraradices* [28]. Some *Mortierella* fungi also produced C20:5N3 [29], while it is not known whether the increase in C20:5N3 in the fungi-colonized trifoliate orange resulted from the production of these fungi in colonized roots. 

In our study, the expression level of *PtΔ9*, *PtΔ15*, *PtFAD2,* and *PtFAD6* in the root was collectively up-regulated by three inoculations with single *Fm*, single *Pi*, and an *Fm* + *Pi* combination, compared with non-fungi inoculation, with the upregulation of single *Pi* being higher in *PtΔ9* and *PtFAD2* than in single *Fm*, and dual inoculation showing a weaker expression pattern. Similar results were observed in our previous study in trifoliate orange after inoculation with *Fm* under ample water conditions [5]. It is a known fact that Δ9 introduced the first double bond into C16:0 and C18:0, and transformed them into C16:1(Δ9) and C18:1 (Δ9) [5]. Although *PtΔ9* was induced by these fungal inoculation, root C16:1 and C18:1 were not increased, and it is possible that root C16:1 and C18:1 were utilized by the endophytic fungi. Δ15 could desaturate C18:2 into C18:3 [30] and, thus, the C18:3 content of roots was increased by *Pi* inoculation, but not by *Fm* and *Fm* + *Pi*, implying a fungi-independent event. FAD2 is located in the endoplasmic reticulum and is responsible for the synthesis of all unsaturated glycerides [31], and FAD6 is distributed on the plastid membrane responsible for the further desaturation of membrane lipid of the plastid membrane [32]. However, an increase in part UFAs was found only in *Pi*-inoculated roots. In fact, spores of AM fungi had the presence of FAs, such as palmitoleic, palmitic, and oleic acids [33,34]. Thus, AM fungi are able to synthesize the lipids from the sugars that they receive from the host plant to sustain their growth [35]. On the other hand, arbuscular mycorrhizas receive FAs from their host plant [13]. The change in FA components and the FA desaturase enzyme gene expression in AM fungi-colonized roots was very complicated, due to the inconsistency of variation and the large number of FAs in plants. In addition, the root material in this study was collected 140 days after endophytic fungal colonization, and many of the fungi-induced lipid changes had already occurred [36]. Therefore, a future analysis of changes within 1–2 weeks of endophytic fungal inoculation may better elucidate the relationship between endophytic fungi and root lipids.

## 5. Conclusions

*Fm* displayed a stronger ability than *Pi* to colonize the roots of trifoliate orange seedlings, but *Pi* caused more accumulation of carbohydrate in leaves and roots and a higher expression level of *PtFAD2* and *PtΔ9* than *Fm*. *Pi* also triggered stronger changes in the unsaturated and saturated fatty acids content than *Fm*, coupled with the collective increase in C14:0 and C20:5N3. These observations also put forth a fact that the root endophytic fungus *Pi* had stronger responses of sugars and fatty acids than *Fm*, implying differently symbiotic mechanisms between the two fungi. Such results provide a better pathway for the future application of *Pi* (an in vitro culturable fungus) to citriculture than *Fm* (a fungus that is difficult to grow in vitro).

## Figures and Tables

**Table 1:** Primer sequences of selective genes.

Genes	Gene IDs	Sequence (5’→3’)-Forward	Sequence (5’→3’)-Reverse
*PtFAD2*	Orange1.1t02241	AGGAGGCAAGAGTGGAGGATAAGG	GGAGCAGGTGGACGAATGTCTG
*PtFAD6*	Cs8g17450	CTGCACGGAGATACAGCTTGGC	GGAATGTGAGGAGCCGTATGATGC
*PtΔ9*	orange1.1t03533	TGCCTGCTCACTTGATGTACGATG	CTCCTCCAGCCTTCTGATTCTTGC
*PtΔ15*	Cs6g08600	CAAGAACTGGTCTAGCAGCCTCAG	ATGTGGCTGGACCTTGTGACTTAC
*β-Actin*	Cs1g05000	CCGACCGTATGAGCAAGGAAA	TTCCTGTGGACAATGGATGGA

**Table 2:** Effects of *Piriformospora indica* (*Pi*) and *Funneliformis mosseae* (*Fm*) singly and in combination on tissue concentration (mg/g DW) of sucrose, glucose, and fructose in trifoliate orange seedlings.

Treatments	Sucrose		Glucose		Fructose	
	Leaves	Roots	Leaves	Roots	Leaves	Roots
Control	58.86 ± 6.57 c	25.54 ± 4.61 b	66.30 ± 6.41 c	86.16 ± 27.40 b	112.81 ± 4.96 c	114.80 ± 8.03 b
*Fm*	73.47 ± 4.77 b	34.95 ± 7.82 b	155.45 ± 3.48 ab	125.42 ± 7.68 a	125.76 ± 2.61 ab	138.09 ± 1.96 a
*Pi*	83.11 ± 3.63 a	57.25 ± 9.56 a	171.42 ± 34.44 a	141.84 ± 9.15 a	130.50 ± 0.40 a	141.34 ± 5.56 a
*Fm* + *Pi*	66.55 ± 2.43 b	52.01 ± 11.68 a	85.43 ± 7.88 c	129.45 ± 12.77 a	123.56 ± 3.13 b	132.53 ± 7.15 a

Data (means ± SD, *n* = 4) followed by different letters in the column indicate significant differences (*p* < 0.05).

**Table 3:** Effects of *Piriformospora indica* (*Pi*) and *Funneliformis mosseae* (*Fm*) singly and in combination on the root fatty acid composition of trifoliate orange seedlings.

FA Types	FA Species	Treatments (μg/g FW)			
		Control	*Pi*	*Fm*	*Fm* + *Pi*
Saturated FAs	Methyl hexanoate (C6:0)	1.47 ± 0.48 b	2.13 ± 0.30 a	1.96 ± 0.17 ab	1.63 ± 0.09 ab
	Methyl octanoate (C8:0)	0.53 ± 0.05 b	0.92 ± 0.21 a	0.50 ± 0.02 b	0.45 ± 0.01 b
	Methyl decanoate (C10:0)	1.18 ± 0.17 ab	1.44 ± 0.20 a	1.04 ± 0.08 bc	0.89 ± 0.06 c
	Methyl undecanoate (C11:0)	ND	ND	ND	ND
	Methyl laurate (C12:0)	8.80 ± 0.57 a	9.56 ± 0.69 a	8.06 ± 1.27 ab	7.31 ± 0.43 b
	Methyl tridecanoate (C13:0)	1.32 ± 0.14 b	1.48 ± 0.19 a	1.39 ± 0.07 ab	1.30 ± 0.14 ab
	Methyl myristate (C14:0)	18.14 ± 0.90 c	23.80 ± 1.70 a	21.15 ± 1.10 b	17.76 ± 1.18 c
	Methyl pentadecanoate (C15:0)	11.38 ± 0.90 a	13.86 ± 0.63 a	12.84 ± 1.51 a	11.30 ± 1.85 a
	Methyl palmitate (C16:0)	1210.76 ± 114.10 a	1379.62 ± 172.93 a	1274.72 ± 14.76 a	1227.51 ± 22.79 a
	Methyl heptadecanoate (C17:0)	16.98 ± 2.19 a	16.91 ± 1.59 a	17.07 ± 3.18 a	15.93 ± 2.23 a
	Methyl stearate (C18:0)	767.37 ± 124.56 a	823.26 ± 28.44 a	791.65 ± 22.28 a	757.79 ± 30.67 a
	Methyl arachidate (C20:0)	12.89 ± 2.34 ab	13.69 ± 0.82 a	12.01 ± 0.13 ab	10.80 ± 0.64 b
	Methyl heneicosadienoate (C21:0)	2.11 ± 0.19 a	2.14 ± 0.14 a	1.92 ± 0.20 ab	1.72 ± 0.22 b
	Methyl behenate (C22:0)	13.62 ± 1.36 ab	15.82 ± 1.68 a	13.46 ± 1.90 ab	12.02 ± 1.16 b
	Methyl tricosanoate (C23:0)	13.22 ± 1.43 ab	15.54 ± 1.74 a	12.16 ± 1.84 b	10.70 ± 1.01 b
	Methyl lignocerate (C24:0)	26.45 ± 3.96 b	36.00 ± 5.54 a	28.76 ± 5.06 ab	26.88 ± 3.24 b
Unsaturated FAs	Methyl myristoleate (C14:1)	ND	ND	ND	ND
	Methyl pentadecenoate (C15:1)	ND	ND	ND	ND
	Methyl palmitoleate (C16:1)	9.97 ± 2.21 a	9.15 ± 1.83 a	7.61 ± 2.99 a	8.90 ± 1.47 a
	Methyl heptadecenoate (C17:1)	8.45 ± 1.77 a	9.09 ± 0.32 a	9.59 ± 0.34 a	8.47 ± 0.15 a
	Methyl oleate (C18:1)	175.21 ± 20.81 a	162.46 ± 25.39 ab	158.08 ± 2.69 ab	125.60 ± 20.85 b
	Methyl linoleate (C18:2)	548.39 ± 40.90 a	542.91 ± 42.95 a	490.88 ± 90.61 a	459.15 ± 29.9 a
	Methyl gamma-Linolenate (C18:3N6)	6.51 ± 0.56 b	10.66 ± 1.35 a	7.49 ± 2.45 b	8.24 ± 0.39 ab
	Methyl linolenate (C18:3N3)	149.23 ± 21.49 a	159.62 ± 16.98 a	133.37 ± 13.39 ab	115.82 ± 6.63 b
	cis-11-Eicosenoic acid methyl ester (C20:1)	8.68 ± 0.82 ab	9.90 ± 1.01 a	8.96 ± 0.13 ab	7.84 ± 0.25 b
	cis-11,14-Eicosadienoic acid methyl ester (C20:2)	2.31 ± 0.27 b	3.48 ± 0.77 a	2.79 ± 0.46 ab	2.75 ± 0.34 ab
	Cis-11,14,-Eicosatrienotic acid methyl ester (C20:3N6)	15.61 ± 0.78 b	31.62 ± 6.99 a	18.98 ± 2.30 b	19.13 ± 1.19 b
	Arachidonate (C20:4N6)	34.44 ± 2.96 b	46.50 ± 6.40 a	27.85 ± 6.81 b	28.58 ± 0.01 b
	Cis-11,14,17-Eicosatrienoate acid methyl ester (C20:3N3)	0.81 ± 0.08 b	1.03 ± 0.07 a	0.92 ± 0.05 ab	0.82 ± 0.01 b
	Cis-5,8,11,14,17-eicosapentaenoate tic acid methyl ester (C20:5N3)	1.56 ± 0.43 c	4.53 ± 0.33 b	4.02 ± 1.58 b	6.721 ± 1.58 a
	Methyl erucate (C22:1N9)	8.68 ± 1.90 b	12.23 ± 0.86 a	11.22 ± 1.72 ab	10.99 ± 2.15 ab
	Cis-13,16-Docosadienotic acid methyl ester (C22:2)	ND	ND	ND	ND
	Cis-4,7,10,13,16,19-Docosahexaenotic acid methyl ester (C22:6N3)	ND	ND	ND	ND
	Methyl cis-15-tetracosenoate (C24:1)	15.84 ± 2.56 b	26.73 ± 6.12 a	17.76 ± 2.96 b	18.05 ± 1.99 b

Data (means ± SD, *n* = 4) followed by different letters in the row indicate significant differences (*p* < 0.05). ND, no detected corresponding FA.

## Data Availability

All the data supporting the findings of this study are included in this article.
